# Two new species of *Codonoboea* (Gesneriaceae) from Kenaboi State Park, Peninsular Malaysia

**DOI:** 10.3897/phytokeys.165.56955

**Published:** 2020-10-28

**Authors:** Suhaimi Syahida-Emiza, Yen Yen Sam, Mat Yunoh Siti-Munirah

**Affiliations:** 1 Forest Research Institute Malaysia, 52109 Kepong, Selangor, Malaysia Forest Research Institute Malaysia Selangor Malaysia

**Keywords:** *Codonoboea kenaboiensis*, *Codonoboea ruthiae*, conservation status, endemic, Negeri Sembilan

## Abstract

Two new *Codonoboea* species, *C. kenaboiensis* Syahida-Emiza, Y.Y.Sam & Siti-Munirah and *C. ruthiae* Syahida-Emiza, Y.Y. Sam & Siti-Munirah were discovered from the Kenaboi State Park, Peninsular Malaysia. Descriptions, illustrations, colour plates and provisional conservation status are provided.

## Introduction

Kenaboi State Park is the first and only state park in Negeri Sembilan, Peninsular Malaysia. The park is located within the greater Kenaboi Forest Reserve which lies at the south end of the Titiwangsa Range, Peninsular Malaysia’s granite mountain range. With 9,036 ha of tropical evergreen rain forest, the park includes pristine lowland dipterocarp and bamboo forests at low elevation with seraya ridges and hill dipterocarp forests at the higher elevation (Latiff and Faridah-Hanum 2014). The highest peak in Negeri Sembilan, Gunung Besar Hantu, at 1,462 m and the tallest waterfall in Negeri Sembilan, known as Lata Kijang falls 115 m, are both located within the park. Three main rivers, namely Sungai Kenaboi, Sungai Semong, and Sungai Kering and their tributaries flow through the reserve (Ramli et al. 2009) supplying clean water to the states of Negeri Sembilan and Selangor. Before the park was established, part of the forest was classified as water catchment, recreational, educational and wildlife forest in accordance with the National Forestry Act 1984.

In 2010, a scientific expedition to Gunung Besar Hantu was carried out to document the biodiversity at high elevation about 1,400 m. Seven years later in 2017, another expedition called the Lembah Jemaloi Scientific Expedition covered the remaining areas at lower elevations, especially the valley at Lembah Jemaloi. During the expeditions, two unknown species of *Codonoboea* were discovered in the lowland forest below 400 m elevation. After careful examination, the collections were shown to have a unique combination of characters that do not match any existing described species and hence, both are described here as new.

*Codonoboea kenaboiensis* Syahida-Emiza, Y.Y.Sam & Siti-Munirah is a small rheophyte discovered on the rocky riverbank of Sungai Kenaboi. This species is unique in its narrowly elliptic leaves with serrate margins. The plants are usually overshadowed by other larger species and are inconspicuous unless their purplish tubular flowers are blooming. On the other hand, *Codonoboea ruthiae* Syahida-Emiza, Y.Y.Sam & Siti-Munirah was found on the forest floor near one of the tributaries of Sungai Kenaboi. Its distant pairs of unequal leaves and attractive maroon flowers distinguish it from other ground flora.

Peninsular Malaysia is the centre of diversity for the genus *Codonoboea*, the most speciose genus in the Gesneriaceae family of Peninsular Malaysia, with at least 95 species recorded so far (Kiew and Lim 2011, 2019; Lim and Kiew 2014). New taxa continue to be discovered and named as botanical collecting ventures into unexplored localities (Kiew and Lim 2019). *Codonoboea* is commonly found in primary forest from lowlands up to mountains growing on granite, sandstone and quartz-derived soils or rocks (Kiew and Lim 2011). Its distribution ranges from Peninsular Thailand, Sumatra, Singapore, Batam and Lingga Islands, Borneo, Palawan (the Philippines), Sulawesi and New Guinea (Lim and Kiew 2014).

## Taxonomy

### Codonoboea kenaboiensis

Taxon classificationPlantae LamialesGesneriaceae

Syahida-Emiza, Y.Y.Sam & Siti-Munirah sp. nov.

798D622B-7AF8-5A2F-AA1F-3FB5BC3A1AD4

urn:lsid:ipni.org:names:77212567-1

Figs 1 , 2 , Map 1

#### Diagnosis.

Similar to *Codonoboea rheophytica* Kiew in its rheophytic habit, distinct narrow leaves, serrate leaf margin and numerous lateral veins but it differs in its cymose inflorescence with 2–3 flowers (*C. rheophytica* has one-flowered inflorescences), small tubular flowers (1–1.3 cm vs. 3–3.5 cm long) and flower colour, violet not white as in *C. rheophytica*.

**Figure 1.**
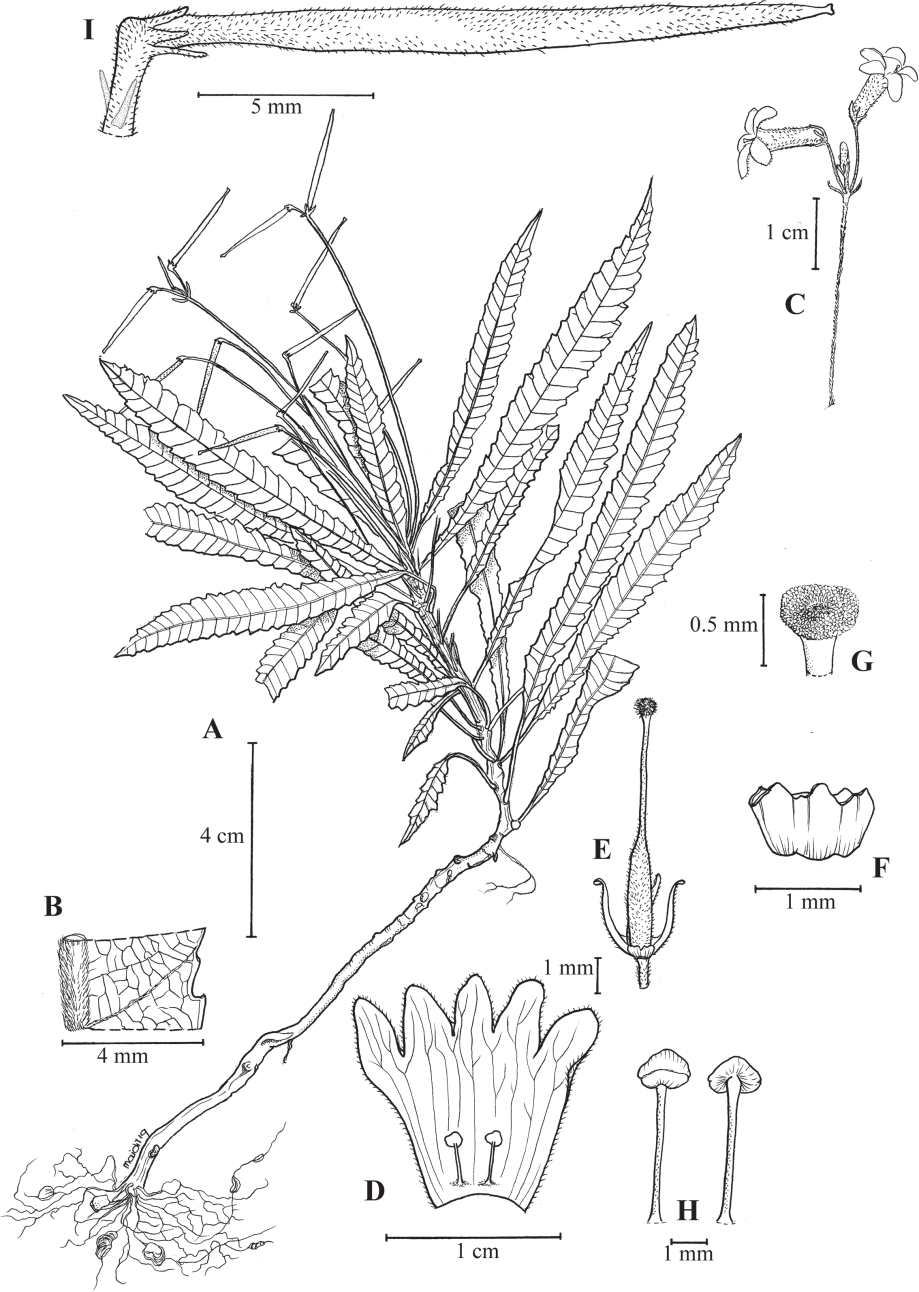
Illustration of *Codonoboea kenaboiensis* Syahida-Emiza, Y.Y.Sam & Siti-Munirah, sp. nov. **A** habit **B** leaf section show midrib and reticulate vein **C** inflorescence **D** flower opened to show position of stamens **E** calyx with pistil **F** nectary annular **G** stigma **H** stamens **I** fruit (All from *FRI 87630*, drawn by Mohamad Aidil Noordin).

#### Type.

Peninsular Malaysia. Negeri Sembilan: Jelebu, Kenaboi Forest Reserve, 3°10'N, 101°59'E, 11 July 2019, Syahida Emiza, S., Sam, Y.Y. & Angan, A. FRI 87630 (holotype: KEP; isotype: K, SING).

**Figure 2.**
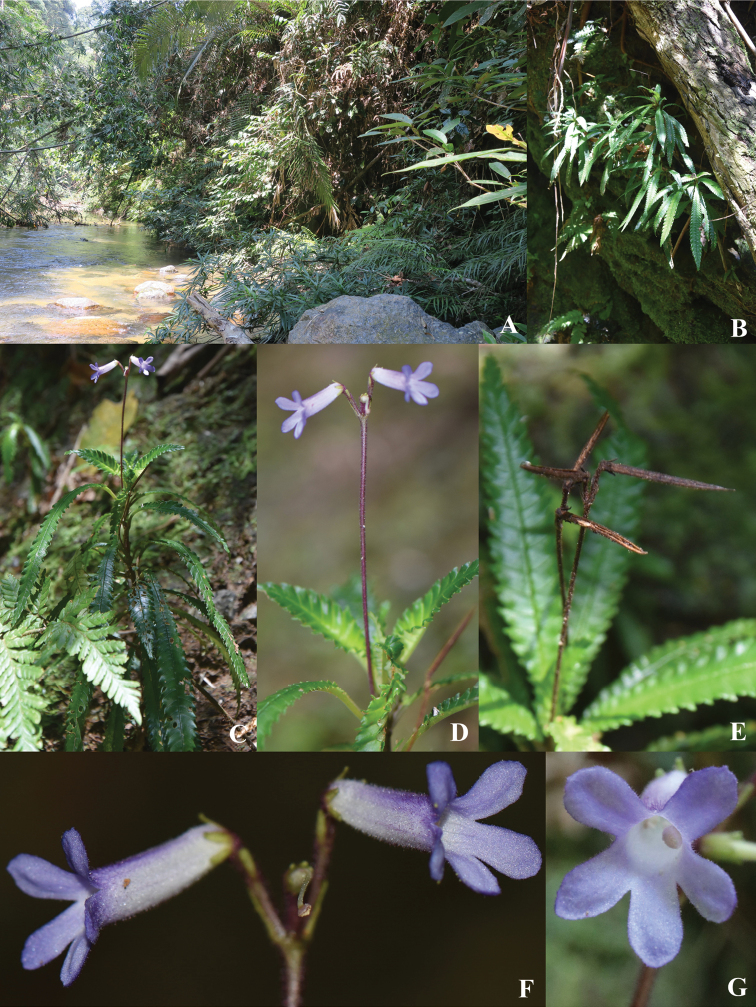
*Codonoboea kenaboiensis* Syahida-Emiza, Y.Y.Sam & Siti-Munirah, sp. nov. **A, B** habitat **C, D** habit **E** fruits **F** flowers **G** corolla lobes (front view).

#### Description.

Rheophyte, 10–20 cm tall. ***Stems*** woody, erect, wiry, unbranched. ***Leaves*** decussate, pairs spaced up to 0.5 cm apart, denser to the apex; petioles slender, 0.5–1 cm long, *c.* 0.1 cm diameter; laminas narrowly elliptic, 4.5–10 × 0.8–1 cm, coriaceous, mid-green above, pale green beneath, base attenuate, apex acute to attenuate, margin serrate; midrib and lateral veins sunken above, glabrous, prominent beneath, puberulous, lateral veins up to 30 pairs, generally opposite or alternately arranged, intercostal veins reticulate, slightly prominent. ***Inflorescences*** at upper leaf axils, erect, 1 per axil, cymes, 2–3-flowered; indumentum of floral parts a combination of glandular and simple hairs; peduncle 4–8 cm, purplish-maroon, densely hairy; bracts 3–3.5 × c. 1 mm, densely hairy, lanceolate, apex blunt. ***Flowers***: pedicels 2–3 mm long, purplish, densely hairy; calyx mid-green, densely hairy, 5-lobed, narrowly lanceolate, lobes 1.2–1.5 × 0.2–0.8 mm, apex blunt; corolla tubular, 10–13 mm long, base 2 mm wide, dilating to 3 mm at the mouth, outside whitish to violet, sparsely hairy, inside whitish, glabrous, veins conspicuous; corolla lobes deeper violet, unequal in size, oblong, *c.* 3 × 2 mm, apex blunt; stamens 2, 3–3.5 mm long, filaments erect, *c.* 2.5 mm long, glabrous, anthers versatile, *c.* 0.8 mm long, *c.* 1 mm wide, white; nectary annular, *c.* 0.6 mm tall, rim toothed, glabrous; pistil yellowish-cream, *c.* 8 mm long, ovary *c.* 3.5 × 0.7 mm, sparsely hairy, style *c.* 4.5 mm long, white, sparsely hairy, stigma peltate, *c.* 0.7 mm across, papillose. ***Capsules*** cylindrical, 18–20 mm long, *c.* 1 mm wide at base, green, splitting adaxially when mature, calyx persistent. ***Seeds*** elliptic-oblong, *c.* 0.2 × 0.15 mm.

#### Other specimen examined.

Peninsular Malaysia. Negeri Sembilan: Jelebu, Kenaboi Forest Reserve, 6 May 2010, Mohd. Hairul, M.A. et al. FRI 70988 (KEP).

#### Distribution.

Endemic in Negeri Sembilan, Peninsular Malaysia. Currently known only from the type locality.

**Map 1.**
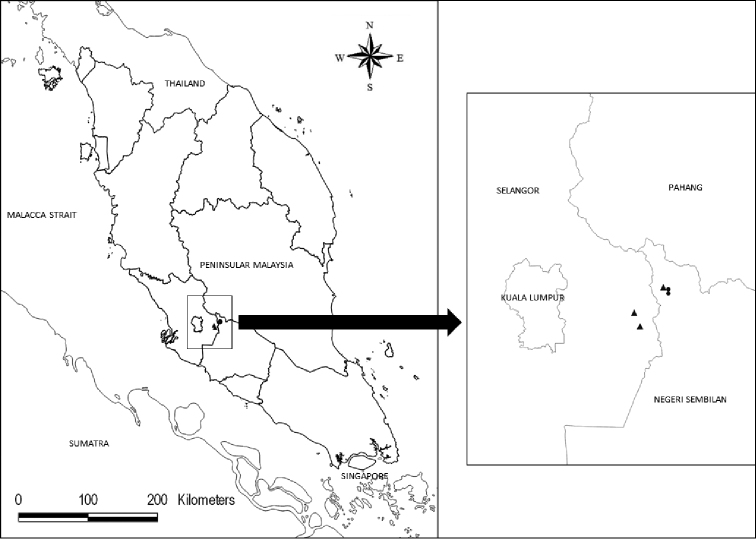
Distribution of *Codonoboea kenaboiensis* (circle) and *Codonoboea ruthiae* (triangle) in Peninsular Malaysia.

#### Ecology.

Lowland dipterocarp forest, on rocks or steep earthy river banks, at 275–315 m altitude. Flowering from May to July. *Codonoboea kenaboiensis* grows on the lower levels of the riparian zone to about 1.5 m above the normal water level. Conditions are cool and damp with a thick layer of mosses covering the ground and shrubs and trees on higher ground leaning towards the river providing partial shade to the vegetation underneath. Such conditions are ideal for many plants but they are subjected to regular flooding events such as flash floods and annual floods during monsoon seasons. Only plants with rheophytic adaptations like *C. kenaboiensis* can survive the swift moving flood water. It has narrow leaves and roots that secure it firmly to the ground, preventing it from being uprooted by water currents. However, more frequent and intense flooding as a result of climate change might affect its long-term survival at Sungai Kenaboi.

#### Etymology.

The epithet refers to the Kenaboi State Park, its only known locality.

#### Conservation status.

Vulnerable, VU D2. *Codonoboea kenaboiensis* is hyper- endemic with a very small and restricted population at Sungai Kenaboi, Kenaboi State Park. Its small population and position in the flood zone is threatened by the increasing records of flood and extreme weather events caused by climate change. Tang (2019) has projected more rainfall events of high intensity and tropical storms in Malaysia as climate change intensifies. Under such circumstances, the seeds and seedlings of *C. kenaboiensis* will be washed away before they can firmly establish to the substrate thereby affecting its regeneration. Following the IUCN Standards and Petitions Committee (2019), *C. kenaboiensis* qualifies for VU D2 because the increased frequency of floods is expected to cause a population reduction and, due to its small population, the species could become Critically Endangered or Extinct in a very short period of time.

#### Discussion.

*Codonoboea kenaboiensis* belongs to Codonoboea sect. Pectinati, characterised by narrow and serrate to deeply toothed leaves and small tubular flower (Ridley 1923; Lim and Kiew 2014). It closely resembles *C. rheophytica*, a recently described rheophyte from Terengganu (Kiew and Lim 2019). Both have distinctly narrow leaves (0.8–1 cm in *C. kenaboiensis* and 0.8–1.3 cm in *C. rheophytica*), serrate leaf margins and numerous lateral veins (30 pairs in *C. kenaboiensis* and 33–36 pairs in *C. rheophytica*). However, they are distinguished by the inflorescence and floral structures. *Codonoboea kenaboiensis* has simple cymes bearing 2–3 tubular flowers whereas *C. rheophytica* has solitary trumpet-shaped flowers. In addition, the violet flowers of *C. kenaboiensis* are much smaller (*c.* 1–1.3 cm vs 3–3.5 cm in *C. rheophytica*) than the white flowers of *C. rheophytica*.

*Codonoboea* species are common on stream and river banks but very few are true rheophytes. Other than *C. rheophytica* and *C. kenaboiensis*, *C. densifolia* (Ridl.) C.L.Lim and *C. salicina* (Ridl.) C.L.Lim are two other rheophytes found in Peninsular Malaysia (Kiew 1987). The narrow leaves of *C. densifolia* and *C. salicina* have entire to serrulate margins and fewer lateral veins (up to 20 pairs) that clearly distinguish them from *C. kenaboiensis*. In terms of inflorescence structure, both *C. densifolia* and *C. salicina* have cymose inflorescences similar to *C. kenaboiensis* but their campanulate flowers are distinct from the tubular flowers of *C. kenaboiensis*.

**Figure 3.**
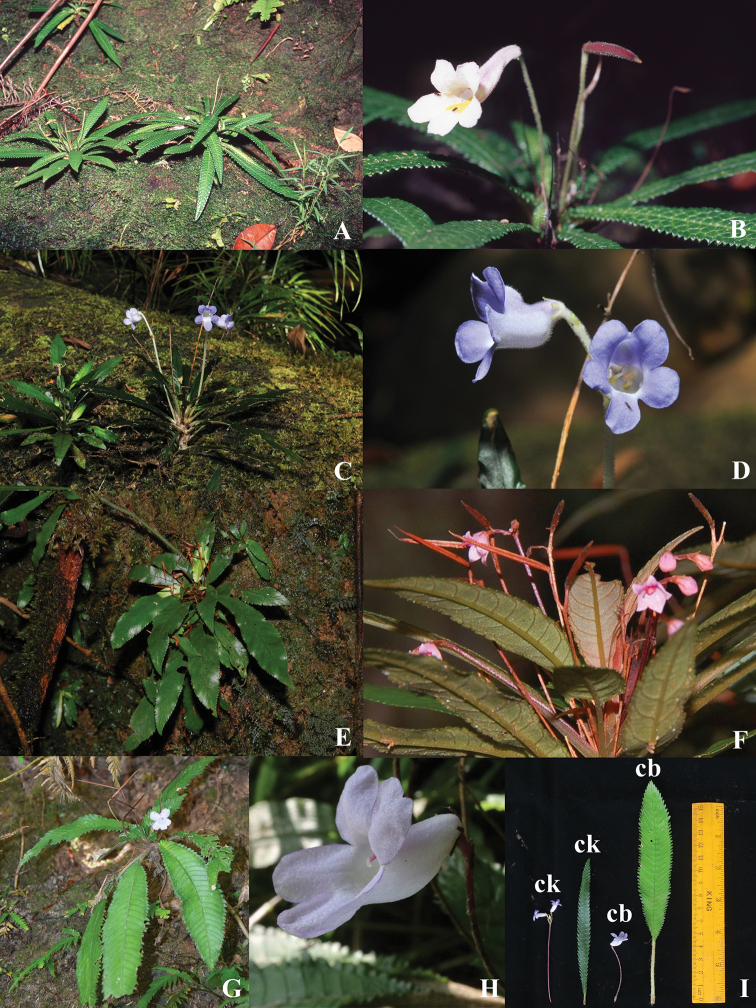
**A, B***Codonoboea rheophytica***C, D***Codonoboea densifolia***E, F***Codonoboea salicina***G, H***Codonoboea breviflora***I** ck = *Codonoboea kenaboiensis*, cb = *Codonoboea breviflora*.

At Sungai Kenaboi, there is another *Codonoboea* species growing on the steep riverbanks. *Codonoboea breviflora* can easily be mistaken as a larger form of *C. kenaboiensis* that also has long narrow leaves and serrate leaf margin. However, the leaves of *C. kenaboiensis* are conspicuously smaller with shorter petioles (0.5–1 cm vs 1–2.5 cm long) and smaller laminas (4.5–10 × 0.8–1 cm vs 9–20 × 2.5–5.5 cm) compared to *C. breviflora* (Fig. 3). Upon closer examination, the leaf venation of *C. kenaboiensis* also proves to be distinct from *C. breviflora*. *Codonoboea kenaboiensis* has craspedodromus lateral veins (the veins run directly from midrib to the margin) whereas in *C. breviflora* the veins branch before reaching the margin. In addition, the single campanulate flower of *C. breviflora* instantly differentiates it from *C. kenaboiensis*.

Both *C. kenaboiensis* and *C. breviflora* grow at Sungai Kenaboi, but based on observations they occupy different sections of the flood zone. *Codonoboea kenaboiensis* resides on the lower levels of the river banks that are less than 1.5 m above the normal water level whereas *C. breviflora* is found at slightly higher levels more than 2 m above the water level. The higher ground is less affected by floods and supports a denser vegetation compare to the site of *C. kenaboiensis*.

### Codonoboea ruthiae

Taxon classificationPlantae LamialesGesneriaceae

Syahida-Emiza, Y.Y.Sam & Siti-Munirah sp. nov.

CFF9D77A-E5D6-5585-8148-864BEDCDA003

urn:lsid:ipni.org:names:77212568-1

Figs 4 , 5 , Map 1

#### Diagnosis.

Amongst species in Codonoboea sect. Didymanthus, *Codonoboea ruthiae* is most similar to *C. ramosa* (Ridl.) Kiew, but can be distinguished by having a larger lamina (9.5–15.5 × 3.5–6.2 cm vs 5.1–7.6 × c. 3.8 cm) with more lateral veins (10–12 vs *c.* 7 pairs); the inflorescences in *C. ruthiae* consists of 5–6 flowers (only 2 in *C. ramosa*) with larger flowers (corolla tube 1.7–1.9 cm long vs c. 1.3 cm long) and flower colour (maroon vs greenish yellow)

**Figure 4.**
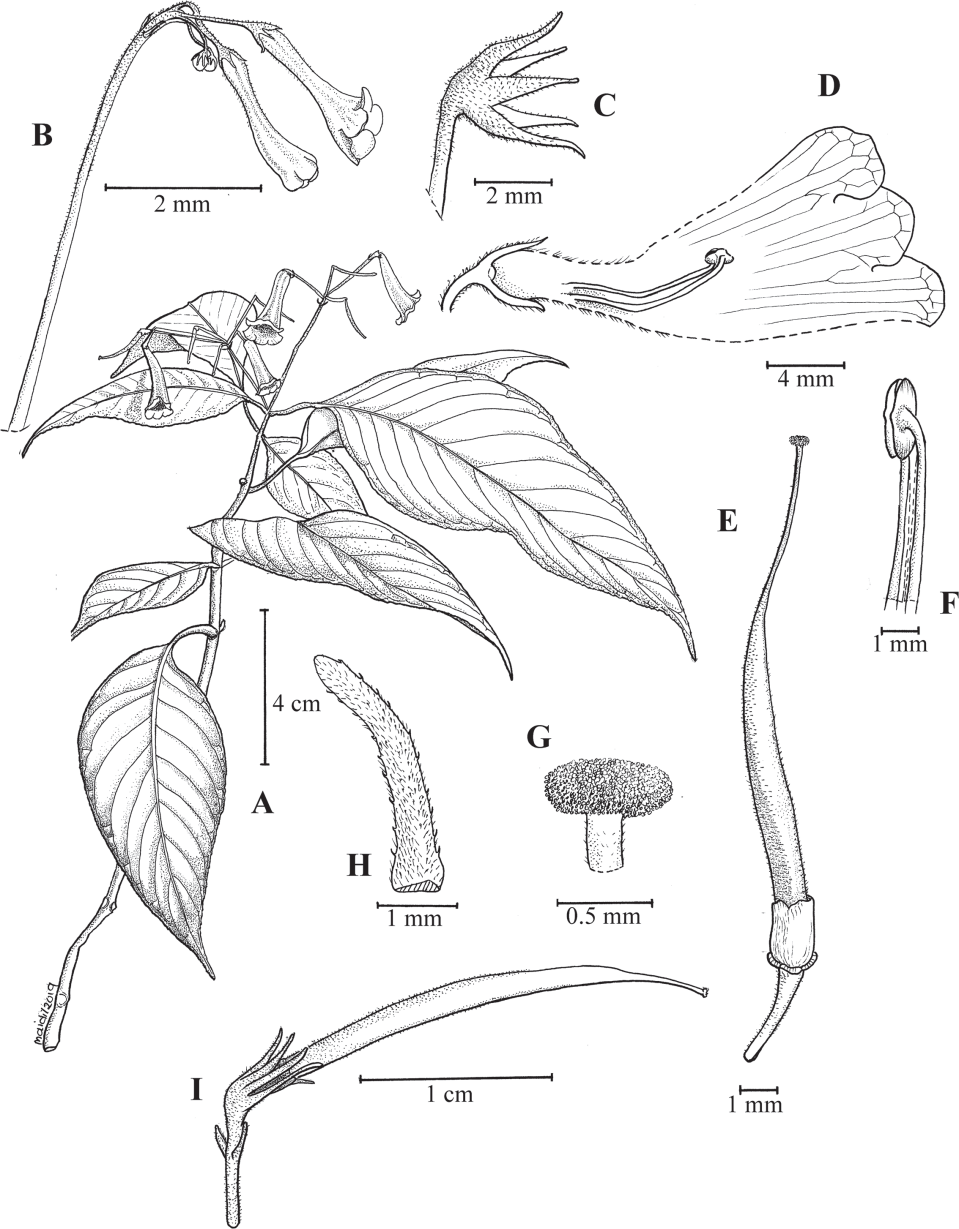
Illustration of *Codonoboea ruthiae* Syahida-Emiza, Y.Y.Sam & Siti-Munirah, sp. nov. **A** habit **B** inflorescence **C** calyx **D** flower opened to show position of stamens **E** pistil **F** anthers **G** stigma **H** bract **I** fruit (All from *FRI 86960*, drawn by Mohamad Aidil Noordin).

#### Type.

Peninsular Malaysia. Negeri Sembilan: Jelebu, Kenaboi Forest Reserve, 3°10'48.6"N, 101°58'19.9"E, 30 October 2017, Syahida Emiza, S., Sam, Y.Y., Yap, J.W., Angan, A. & Markandan, M. FRI 86960 (holotype: KEP)

**Figure 5.**
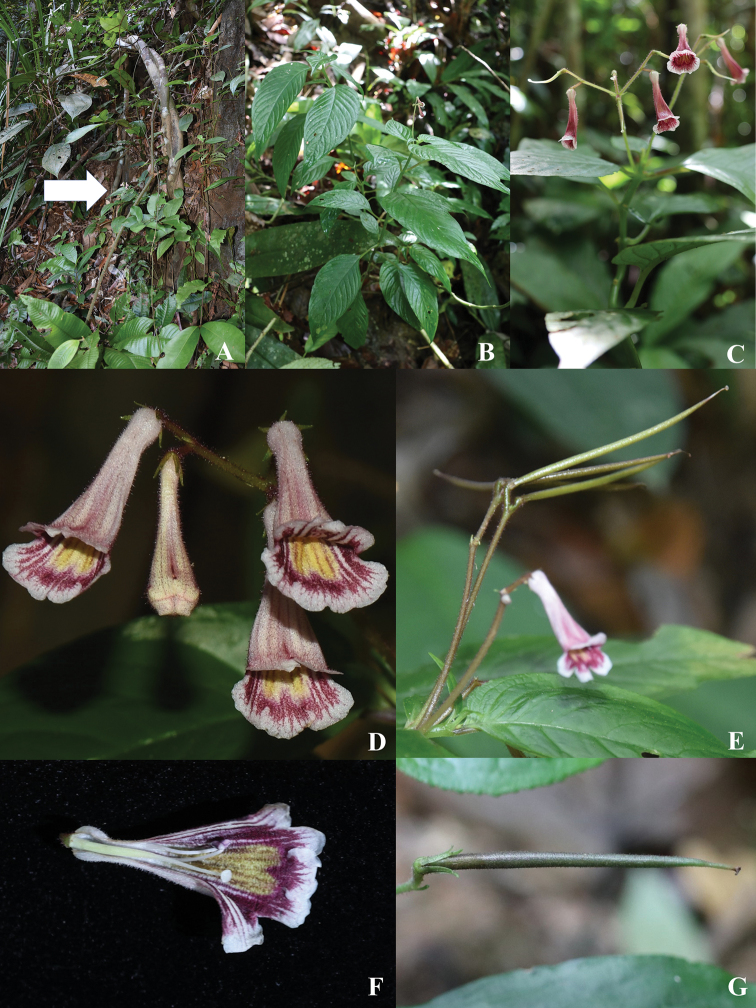
*Codonoboea ruthiae* Syahida-Emiza, Y.Y. Sam & Siti-Munirah. **A** habitat **B** habit **C** inflorescence with flower and pistil (corolla fallen) **D** flowers from front view **E** flower and fruits **F** longitudinal section of flower **G** capsule.

#### Description.

Erect, perennial herb, 20–50 cm tall. ***Stems*** slender-branched, 3–4 mm diameter, semi-woody at base. ***Leaves*** decussate, pairs spaced up to 4.5 cm apart, very unequal; petioles slender, grooved above, pale green, puberulous to glabrescent; smaller leaves less than half the size of larger leaves, petioles 0.5–1.5 cm, *c.* 0.1 cm diameter, laminas 5.5–7.5 × 1.5–3 cm; larger leaf with petioles 1.0–3.2 cm long, *c.* 0.1 cm diameter, laminas lanceolate to ovate, 9.5–15.5 × 3.5–6.2 cm, asymmetric, thinly coriaceous, mid-green above, glabrous, pale green beneath, sparsely puberulous to glabrescent, base oblique, apex acute, margin serrulate, sparsely puberulous; midrib and lateral veins raised on both surfaces, sparsely puberulous to glabrescent, lateral veins 10–12 pairs, alternately arranged, intercostal veins reticulate, slightly prominent. ***Inflorescences*** at upper most leaf axils, erect, pair-flowered cyme twice branched with 5–6 flowers; indumentum of floral parts a combination of glandular and simple hairs, simple hairs 0.75–1 mm long; peduncle 4–5 cm, reddish green, densely hairy; bracts and bracteoles lanceolate, bracts *c.* 3.2 × 0.7 mm, bracteoles *c.* 2.5 × 0.5 mm. ***Flowers***: pedicels 2–10 mm long, greenish maroon, densely hairy; calyx mid-green, densely hairy, 5-lobed, calyx lobes three quarters of the calyx length, lobes narrowly acute, 2.1–2.5 × 0.4–0.5 mm, apex slightly blunt; corolla trumpet-shaped, 20–24 mm long, base narrow, 1–1.5 mm wide, dilating to 7–8 mm wide at mouth, tube 17–19 mm long, veins conspicuous, outside pale maroon, sparsely hairy and hirsute, inside with deep maroon streaks, throat with two yellow nectar guides, veins conspicuous, pubescent from base up to stamens attach, upper lobes nearly rounded, *c.* 4 × 5 mm, slightly reflexed, side lobes rounded *c.* 5 × 5 mm, lower lobe near transverse elliptic, *c.* 5 × 7 mm; stamens 2; filaments slender, *c.* 5.3 mm long, glabrous, slightly curved upward, attached to corolla tube at *c.* 7 mm from base, anthers reniform, small, *c.* 2 mm long, 0.6 mm wide, white, dorsifixed, fused face-to-face; nectary annular, *c.* 1.3 mm tall, rim undulate, glabrous; pistil whitish-cream, *c.* 13 mm long, ovary 7–8 × c. 0.6 mm, glabrous; style 5–6 mm long, white, sparsely hirsute; stigma peltate, *c.* 0.8 mm across, papillose. ***Capsules*** cylindrical, very slender, 25–40 mm long, 1–1.5 mm wide, maroon-green, densely hairy, splitting adaxially when mature; calyx persistent. ***Seeds*** elliptic-oblong, *c.* 0.3 mm × 0.25 mm.

#### Other specimens examined.

Peninsular Malaysia. Selangor: Hulu Langat, Sungai Lalang Forest Reserve, Compartment 42, 29 June 2004, Sam Y.Y. et al. FRI 47248 (KEP), Compartment 43, 13 February 2019, Sam Y.Y. et al. FRI 69261 (KEP, K, L, SAN, SAR, SING).

#### Distribution.

Peninsular Malaysia, recorded in Negeri Sembilan and Selangor.

#### Ecology.

In lowland dipterocarp forest, on forest floor, hill side or slope near shaded small stream at 130–335 m altitude. Flowering in February, June and October.

#### Etymology.

Named after Dr. Ruth Kiew, a prominent botanist, plant taxonomist and conservationist. She is well known for her work on herbaceous plants such as begonias and gesneriads, and also limestone and montane flora.

#### Conservation status.

Least Concern (LC). *Codonoboea ruthiae* is well protected within the Protected Area Network. Its existence in Kenaboi State Park and also at the water catchment forest in Sungai Lalang Forest Reserve, Selangor, which is categorised as a Protection Forest, is legally secured under the National Forestry Act 1984 (Ministry of Water, Land and Natural Resources 2019). Furthermore, no threat to the species population has been identified.

#### Discussion.

*Codonoboea ruthiae* belongs to Codonoboea sect. Didymanthus (Lim and Kiew 2014) characterised by opposite and well-spaced petiolate leaves. It closely resembles *C. ramosa* in its slender-branched stem, distant pairs of leaves, one of each pair much smaller, oblique lamina base and trumpet-shaped flowers (Ridley 1923). However, *C. ruthiae* can be distinguished from *C. ramosa* by a combination of characters shown in Table 1.

**Table 1.** Differences between *Codonoboea ruthiae* and *C. ramosa*.

Character	*Codonoboea ruthiae*	*Codonoboea ramosa*
Lamina size of larger leaf (cm)	9.5–15.5 × 3.5–6.2	5.1–7.6 × c. 3.8
Lateral veins (pairs)	10–12	*c.* 7
Peduncle (cm)	4–5	2.5–3.2
Number of flowers	5–6	2
Flower colour	Maroon	Greenish yellow
Corolla tube (cm)	1.7–1.9	*c.* 1.3

## Supplementary Material

XML Treatment for Codonoboea kenaboiensis

XML Treatment for Codonoboea ruthiae

## References

[B1] Kiew R (1987) The herbaceous flora of Ulu Endau, Johore-Pahang, Malaysia, including taxonomic notes and descriptions of new species. Malayan Nature Journal 41: 201–234.

[B2] Kiew R Lim CL (2011) Names and new combinations for Peninsular Malaysian species of *Codonoboea* Ridl. (Gesneriaceae). Gardens’ Bulletin (Singapore) 62(2): 253–275.

[B3] Kiew R Lim CL (2019) *Codonoboea* (Gesneriaceae) in Terengganu, Peninsular Malaysia, including three new species. PhytoKeys 131: 1–26. 10.3897/phytokeys.131.35944 31537960 PMC6733809

[B4] Latiff A Faridah-Hanum I (2014) Roles and functions of Hutan Simpan Gunung Besar Hantu in biodiversity conservation. In: Abdul Rahman AR Mohd Nasir AH Ahmad Fadzil AM Richard AM Latiff A (Eds) Hutan Gunung Besar Hantu Negeri Sembilan: Pengurusan Hutan, Persekitaran Fizikal dan Kepelbagaian Biologi.Jabatan Perhutanan Semenanjung Malaysia, Kuala Lumpur, 30–37.

[B5] Lim CL Kiew R (2014) *Codonoboea* (Gesneriaceae) sections in Peninsular Malaysia. Reinwardtia 14(1): 13–17. 10.14203/reinwardtia.v14i1.388

[B6] Ministry of Water Land and Natural Resources (2019) A Master List of Protected Areas in Malaysia – A Tool for National Biodiversity Conservation Management and Planning. Ministry of Water, Land and Natural Resources, Putrajaya, 141 pp.

[B7] Ramli R Ya’cob Z Hashim R (2009) Diversity of Birds in Kenaboi Forest Reserve, Jelebu, Negeri Sembilan, Malaysia. Malaysian Journal of Science 28(4): 465–480.

[B8] Ridley HN (1923) Gesneriaceae. Flora Malay Peninsula 2: 495–547. 10.2307/4115416

[B9] IUCN Standards and Petitions Committee (2019) Guidelines for Using the IUCN Red List Categories and Criteria. Version 14. Prepared by Standards and Petitions Committee. http://www.iucnredlist.org/documents/RedListGuidelines.pdf

[B10] Tang KHD (2019) Climate change in Malaysia: Trends, contributors, impacts, mitigation and adaptations. The Science of the Total Environment 650: 1858–1871. 10.1016/j.scitotenv.2018.09.316 30290336

